# The Role of Dual Energy CT in Evaluating Hemorrhagic Complications at Different Stages After Thrombectomy

**DOI:** 10.3389/fneur.2020.583411

**Published:** 2020-10-07

**Authors:** Keqin Liu, Lin Jiang, Jie Ruan, Wenqing Xia, Huan Huang, Guozhong Niu, Shenqiang Yan, Congguo Yin

**Affiliations:** ^1^Department of Neurology, Hangzhou First Hospital, Zhejiang University School of Medicine, Hangzhou, China; ^2^Department of Neurology, School of Medicine, The 2nd Affiliated Hospital of Zhejiang University, Hangzhou, China

**Keywords:** dual energy CT (DECT), ischemic stroke, thrombectomy, hemorrhagic transformation (HT), contrast staining

## Abstract

**Background:** Contrast media extravasation can mimic hemorrhage after endovascular thrombectomy (EVT). Dual energy CT (DECT) has the potential to distinguish hemorrhage from iodine contrast.

**Methods:** We retrospectively examined clinical and radiological data from 106 consecutive acute ischemic stroke patients who received EVT and underwent DECT immediately and 24 h after EVT. Iodine overlay map, virtual non-contrast, and mixed images are reconstructed.

**Results:** With the use of DECT, the proportion of all patients diagnosed with hemorrhagic transformation on mixed images immediately after EVT was reduced from 74.5% (79 of 106) to 10.4% (11 of 106), with a very poor consistency (κ = 0.076, *p* = 0.041). Correspondingly, hemorrhagic transformation on mixed images 24 h after EVT was reduced from 41.5% (44 of 106) to 30.2% (32 of 106), with a moderate consistency (κ = 0.757, *p* < 0.001).

**Conclusions:** The use of DECT both immediately and 24 h after EVT changes the diagnosis of hemorrhagic transformation in a considerable proportion of acute ischemic stroke patients with EVT. This could affect decision making with respect to antithrombotic strategy.

## Introduction

Intravenous thrombolysis (IVT) is an effective and broadly applicable treatment for acute ischemic stroke (1). One of the most important complications of IVT is intracranial hemorrhagic transformation, which is associated with poor outcome and even death (2). Recently, the benefit of endovascular thrombectomy (EVT) has also been established in acute ischemic stroke patients with large artery occlusion (3). Both IV iodine contrast during advanced imaging and intra-arterial injection of iodine contrast during the EVT procedure can lead to contrast staining due to blood-brain barrier breakdown; as a consequence, hyperdense areas were frequently detected on non-contrast CT immediately after EVT (4). Early differentiation between hemorrhage and contrast staining is important for clinical decision making, such as the use of glycoprotein IIb/IIIa inhibitor (tirofiban) in some patients with high risk of early reocclusion after EVT (5), or initiation of secondary preventive treatment with antiplatelet or anticoagulant agents after 24 h (6).

The use of dual energy CT (DECT) might change the radiologic report regarding post-treatment hemorrhagic transformation in a considerable proportion of patients with EVT compared to conventional non-contrast CT (7). In DECT, iodine overlay map (IOM) and virtual non-contrast (VNC) images are reconstructed from two different X-ray spectra at different kilovoltage (kV) either from one X-ray source using kV switching or from two X-ray sources (8, 9). The differentiation between hemorrhage and contrast medium became feasible since the attenuation characteristics of iodine and blood are different at two energy levels.

However, there are few studies trying to verify the diagnostic confidence in differentiation between hemorrhage and contrast medium extravasation after EVT, and to evaluate the clinical value of DECT at different stages. Therefore, in the current study, we aimed to (1) investigate how DECT immediately after EVT changes the diagnosis of hemorrhagic transformation and compare its radiologic report with follow-up CT of 24 h and (2) investigate how DECT 24 h after EVT changes the diagnosis of hemorrhagic transformation and compare its radiologic report with a follow-up CT of 3 days.

## Methods and Materials

### Study Subjects

We retrospectively reviewed our prospectively collected database for consecutive patients with acute ischemic stroke received EVT between January 2016 and October 2018. We then enrolled patients who (i) underwent DECT immediately after EVT; (ii) underwent DECT 24 h after EVT; (iii) underwent conventional non-contrast CT 3 days after EVT. We excluded patients who had recent previous ischemic stroke within 3 months to avoid any potential intracranial hemorrhage and contrast staining findings related to subacute blood-brain barrier breakdown.

### Study Protocols

**Figure 1:**
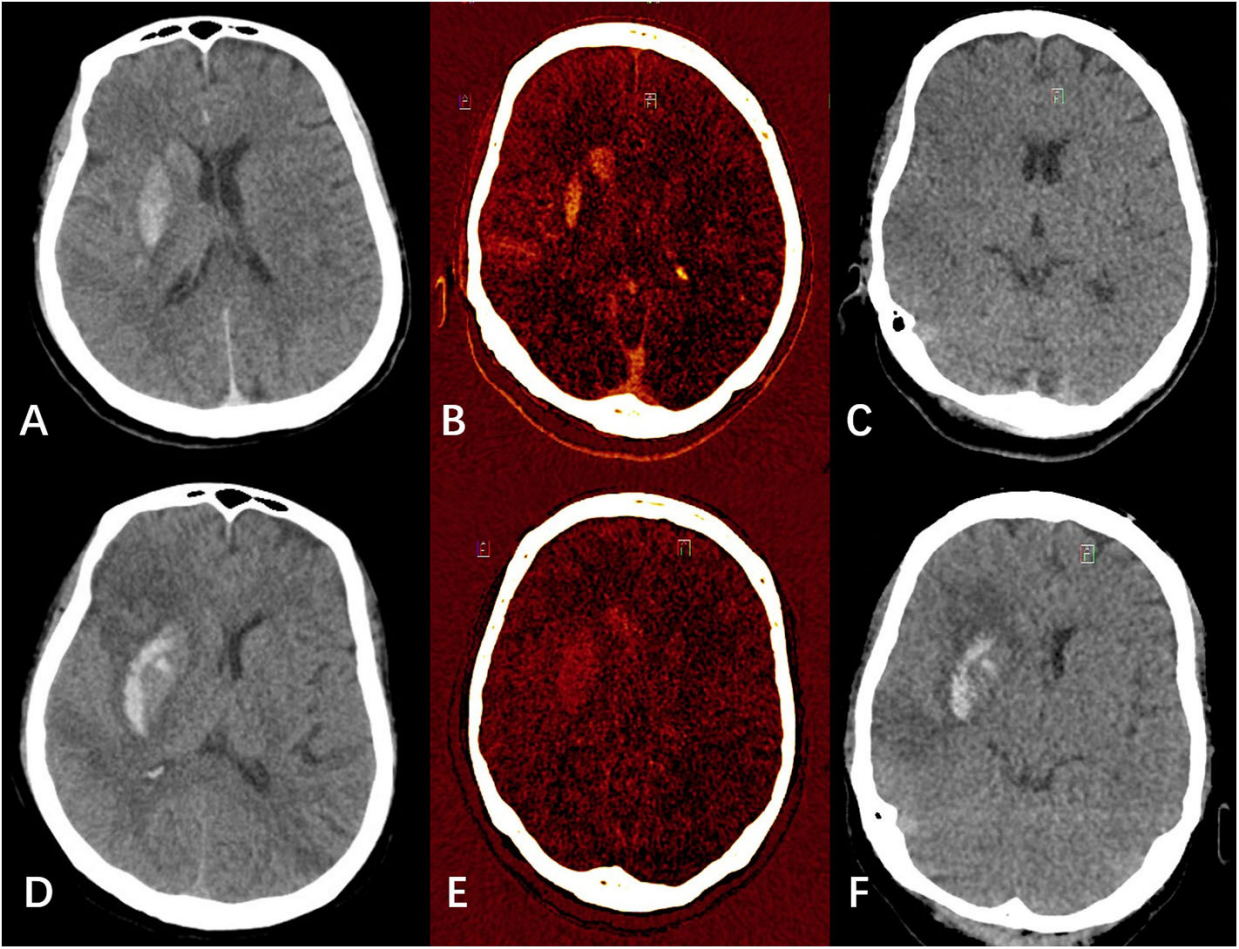
Examples of hemorrhagic transformation and contrast extravasation with iodine overlay map (IOM), virtual non-contrast (VNC), and mixed images. **(A–C)** were mixed image, IOM, and VNC, respectively from a patient's dual energy CT (DECT) immediately after endovascular thrombectomy (EVT). **(A)** showed hyperdensities in the right lentiform nucleus and caudate nucleus. In **(B,C)** combined, the hyperdensities were classified as pure iodine contrast. **(D–F)** were mixed image, IOM, and VNC, respectively from the same patient's DECT 24 h after EVT. **(D)** also showed hyperdensities in the right lentiform nucleus and caudate nucleus. In **(E,F)** combined, the hyperdensities were classified as hemorrhage with iodine contrast.

DECT was performed immediately and 24 h after EVT, and conventional non-contrast CT was performed 3 days after EVT. DECT images were acquired with a dual source 128 slice CT scanner (SOMATOM Force, Siemens Healthcare, Forchheim, Germany). Acquisition and reconstruction of CT parameters were as follows: a dedicated dual-source protocol with simultaneous imaging at 80 kV/392 mAs eff. and 140 kV(Sn)/196 mAs eff., collimation of 0.6 mm and pitch of 0.7 was employed. The raw spiral projection data were rebuilt in three different series, with two sets corresponding to 80 and 140 kV (0.6 mm slice thickness) and a third set corresponding to a mixed map of both energies (80/140 kV), simulating a conventional 120 kV CT. VNC images and IOM were calculated using a dedicated brain hemorrhage algorithm (Syngo; CT Dual-Energy Brain Hemorrhage; Siemens) (Figure 1).

### Image Analysis

Hemorrhagic transformation was classified by using the following radiological criteria: hemorrhagic infarction (HI, including HI-1 and HI-2), parenchymal hemorrhage (PH, including PH-1 and PH-2) (10). DECT images were evaluated for the diagnosis and grading of hemorrhagic transformation. Definitions of contrast material extravasation and intracranial hemorrhage on DECT were previously described in detail elsewhere (11). Mixed images of DECT and conventional non-contrast CT images were evaluated for the presence of hyperdense areas. Hyperdensities were defined as areas with objective higher density than the surrounding brain parenchyma. Hyperdensities visible on mixed images of DECT were interpreted as hemorrhage, contrast extravasation, or both. The interpretations of DECT were compared with conventional non-contrast CT 3 days after EVT. Washout or near-complete clearing of the hyperdensities on follow-up CT was classified as contrast material extravasation, while persisting hyperdensities on follow-up CT were classified as hemorrhage.

Two neurologists with 10 years of experience with acute stroke imaging (KL & LJ) separately reviewed first the mixed images alone and, in a second reading, the IOM and VNC images. Disagreement was resolved by consensus, and in cases with remaining disagreement, the final decision was made by an interventional neurologist (CY).

### Statistical Analysis

The consensus judgment of DECT and conventional non-contrast CT was used as reference. Categorical variables were presented as number and percentage. Continuous variables were summarized as mean ± SD or median with interquartile range (IQR). Significance of difference between proportions was calculated with the Pearson χ^2^ or Fisher's exact test. All analyses were performed blind to participant identifying information.

## Results

Inter-reader agreement of any hemorrhagic transformation immediately after EVT had a κ value of 0.79 on mixed images, and a κ value of 0.76 on VNC images, respectively. Inter-reader agreement of any hemorrhagic transformation 24 h after EVT had a κ value of 0.81 on mixed images, and a κ value of 0.73 on VNC images, respectively. Inter-reader agreement of any hemorrhagic transformation 3 days after EVT had a κ value of 0.71 on conventional non-contrast CT.

A total of 106 remaining patients were included for the final analysis. Of the patients included, 39 (36.8%) were women, with a median age of 74 years (mean 71.6 ± 10.3 years, range 27–86 years). Mean time from onset to puncture was 291.5 (237.3–367.0) min, and mean time from puncture to reperfusion was 50.5 (40.0–82.3) min. The detailed clinical data and demographics are shown in Table 1.

**Table 1:** Clinical characteristics (*n* = 106).

**Variable**	**Mean ± SD or median (IQR) or *n* (%)**
Age (year)	71.6 ± 10.3
Female, *n* (%)	39 (36.8%)
Co-morbid conditions	
Hypertension, *n* (%)	61 (57.5%)
Diabetes mellitus, *n* (%)	13 (12.3%)
Hyperlipidemia, *n* (%)	14 (13.2%)
Atrial fibrillation, *n* (%)	48 (45.3%)
Smoking, *n* (%)	20 (18.9%)
Prior stroke or TIA, *n* (%)	11 (10.4%)
Clinical variables	
NIHSS score	17 (14–20)
Intravenous thrombolysis, *n* (%)	29 (27.4%)
Anterior circulation stroke, *n* (%)	90 (84.9%)
Onset to puncture (min)	291.5 (237.3–367.0)
Puncture to reperfusion (min)	50.5 (40.0–82.3)
Poor clinical outcome, mRS ≥3	51 (48.1%)

*TIA = transient ischemic attack; NIHSS = National Institutes of Health Stroke Scale; mRS = modified Rankin Scale*.

Based on the IOM and VNC images of DECT immediately after EVT (Table 2), 11 patients (10.4%) were classified as hemorrhagic transformation, and all of them were mixed with iodine contrast. In all, 68 patients (64.2%) were classified as pure iodine contrast, while the remaining 27 patients (25.5%) showed no hyperdensities. Based on the IOM and VNC images of DECT 24 h after EVT (Table 2), 32 patients (30.2%) were classified as hemorrhagic transformation, and 17 of them (53.1%) were mixed with iodine contrast. Twelve patients (11.3%) were classified as pure iodine contrast, while the remaining 62 patients (58.5%) showed no hyperdensities.

**Table 2:** Interpretations of hyperdensities on DECT immediately and 24 h after EVT.

	**Immediate DECT**	**24 h DECT**
**Any hemorrhagic transformation**	11 (10.4%)	32 (30.2%)
Hemorrhage with iodine contrast	11 (100.0%)	17 (53.1%)
Hemorrhage without iodine contrast	0 (0.0%)	15 (46.9%)
**Pure iodine contrast**	68 (64.2%)	12 (11.3%)
**No hyperdensities**	27 (25.5%)	62 (58.5%)

*DECT = dual energy CT; EVT = endovascular thrombectomy*.

**Figure 2:**
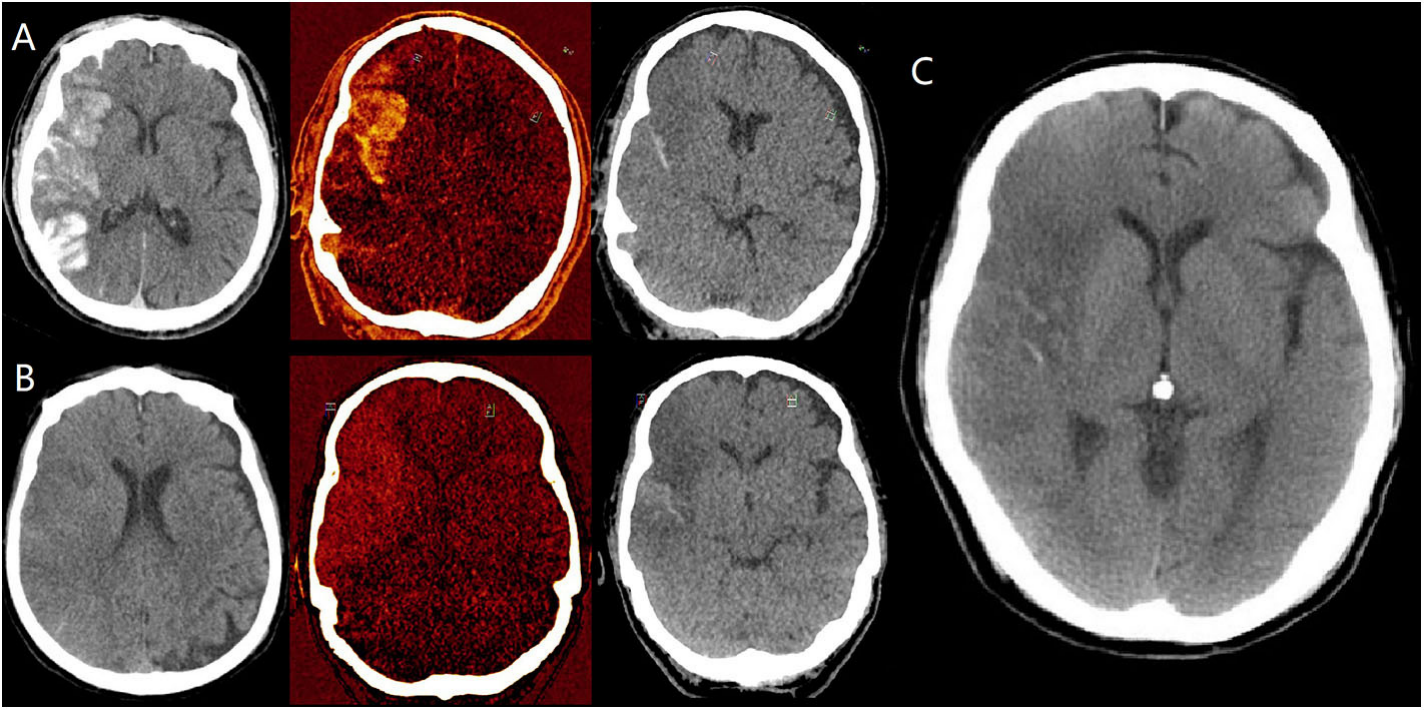
Dynamic changes of hyperdensities in a patient diagnosed with hemorrhagic transformation (mixed with iodine) on dual energy CT (DECT) immediately after EVT. **(A)** are mixed images, iodine overlay map (IOM), virtual non-contrast (VNC) images immediately after EVT, respectively, indicating the presence of both hemorrhage and iodine contrast. **(B)** are also mixed images, iodine overlay map (IOM), virtual non-contrast (VNC) images 24 h after EVT, respectively, indicating the persistent hemorrhage and clearance of iodine contrast. **(C)** is conventional non-contrast CT 3 days after EVT, indicating the partial absorption of hemorrhage.

The 11 patients diagnosed with hemorrhage immediately after EVT (all mixed with iodine) were still shown as hemorrhage on both mixed images 24 h after EVT and non-contrast CT 3 days after EVT, while 5 of them (45.5%) had clearance of iodine contrast based on the DECT 24 h after EVT (Figure 2). The 27 patients with no hyperdensities immediately after EVT remained clean on both mixed images 24 h after EVT and non-contrast CT 3 days after EVT. In 68 patients classified as pure iodine contrast immediately after EVT, 35 of them (51.5%) showed no hyperdensities on mixed images 24 h after EVT, while 12 of them (17.6%) had clearance of iodine contrast on non-contrast CT 3 days after EVT. And the remaining 21 patients (30.9%) developed hemorrhagic transformation on both VNC images 24 h after EVT and non-contrast CT 3 days after EVT.

With the use of DECT, the proportion of all patients diagnosed with hemorrhagic transformation on mixed images immediately after EVT was reduced from 74.5% (79 of 106) to 10.4% (11 of 106), with very poor consistency (κ = 0.076, *p* = 0.041) (Table 3). Correspondingly, the proportion of all patients diagnosed with hemorrhagic transformation on mixed images 24 h after EVT was reduced from 41.5% (44 of 106) to 30.2% (32 of 106), with moderate consistency (κ = 0.757, *p* < 0.001) (Table 3).

**Table 3:** Comparison of hemorrhage judgment on mixed and VNC images.

	**Mixed images**	**VNC images**	**κ**	***p*-value**
**DECT immediately after EVT**			0.076	0.041
Any hemorrhagic transformation	79 (74.5%)	11 (10.4%)		
No hemorrhage	27 (25.5%)	95 (89.6%)		
**DECT 24 h after EVT**			0.757	<0.001
Any hemorrhagic transformation	44 (41.5%)	32 (30.2%)		
No hemorrhage	62 (58.5%)	74 (69.8%)		

*VNC = virtual non-contrast; DECT = dual energy CT; EVT = endovascular thrombectomy*.

The classification of hemorrhagic transformation at different stages after thrombectomy was shown in Table 4. New hemorrhage mostly occurred within 24 h after EVT (from 10.4 to 30.2%). Three patients with no hyperdensities on 24 h DECT developed delayed hemorrhagic transformation (1 HI and 2 PH), while one patient with HI on 24 h DECT became PH on 3 day conventional non-contrast CT.

**Table 4:** Grades of hemorrhagic transformation at different stages after thrombectomy.

	**Immediate VNC**	**24 h VNC**	**3 day NCCT**
**Hemorrhagic transformation grades**			
No hemorrhage	95 (89.6%)	74 (69.8%)	71 (67.0%)
Hemorrhagic infarction	5 (4.7%)	21 (19.8%)	21 (19.8%)
Parenchymal hemorrhage	6 (5.7%)	11 (10.4%)	14 (13.2%)

*VNC = virtual non-contrast; NCCT = non-contrast CT*.

## Discussion

Hyperdense areas were frequently (74.5%) detected on CT immediately after EVT. With the use of DECT, the proportion of patients diagnosed with hemorrhagic transformation immediately after EVT was reduced to 10.4%. Although the phenomenon of contrast medium extravasation became less common at 24 h, the use of DECT still changed the diagnosis of hemorrhagic transformation in a considerable number of patients (11.3%). New hemorrhages mostly occurred within 24 h after EVT (from 10.4 to 30.2%). After excluding some delayed new and progressive hemorrhages, the hemorrhagic transformation classified on 24 h VNC was quite consistent with the 3 day conventional non-contrast CT.

Lummel et al. (12) reported the frequency of hyperdense lesions was 84.2% in patients after EVT, even higher than our current study. Our study showed that 27.2% (12 in 44) of hemorrhage findings in the routine 24 h follow-up group are caused by contrast staining mimicking blood. This proportion increases to 86.1% (68 in 79) in the group scanned immediately after EVT. The former finding is similar to the report from Almqvist et al.'s (7) study of DECT. The latter finding of pure contrast staining proportion is higher than in three previously published studies of a post-interventional DECT strategy within 30, 60, or 120 min (68, 47, and 32%, respectively) (8, 13, 14).

There are several techniques to distinguish contrast staining from hemorrhage. Commonly, the issue can be resolved with a repeated CT examination within 1–3 days (12, 15), which may postpone antithrombotic therapy or anticoagulation. Although iodine can affect several magnetic resonance sequences (16), hemosiderin-sensitive sequences can be used since it is unlikely that iodine could mimic hemorrhage on magnetic resonance imaging (17). By contrast, DECT is a simple and fast solution differentiating hemorrhage and contrast staining, avoiding the delayed time for a repeat examination and the limitations of magnetic resonance, such as contraindications and limited resource issues (7).

Early differentiation between hemorrhage and contrast medium extravasation immediately after EVT is important for clinical decision making, such as whether to start treatment with glycoprotein IIb/IIIa inhibitor (tirofiban) after EVT to prevent early reocclusion due to endothelial damage (5), or might be useful where repeat intervention is necessary. On the other hand, the AHA/ASA guidelines for the management of acute ischemic stroke patients recommended obtaining follow-up imaging 24 h after IVT before starting antiplatelets or anticoagulants (6). However, the high occurrence of hyperdensities on CT after EVT brought concerns to clinicians about the use of antithrombotic agents for secondary preventive treatment, since contrast staining could mimic hemorrhage. Based on the current study, the use of DECT might in our opinion provide this differentiation.

Limitations include a retrospective design in a single stroke center, though we prospectively collected data using a stroke registry, which might present a potential risk of selection bias. Some severe stroke patients might be transferred to an intensive care unit or receive surgical treatment the next day making them unable to undergo follow-up DECT within 24 h. The sample size is moderate; future large multicenter studies and individual patient data meta-analysis are needed to further investigate the importance of DECT in the patients with EVT. On the other hand, the best verification of our confidence in DECT's diagnostic differentiation between hemorrhage and contrast medium extravasation is by performing hemosiderin-sensitive magnetic resonance sequences at the same time, which is clinically difficult. Considering the clearance of iodine contrast, we used a conventional non-contrast CT 3 days after EVT for the verification, although a few patients developed new or progressive hemorrhagic transformation.

Standard non-contrast CT alone should be used with caution for the diagnosis and grading of hemorrhagic transformation after EVT, because contrast staining can mimic hemorrhage. We concluded that DECT with IOM and VNC has potential and is essential for early differentiation of hemorrhage and contrast material extravasation after EVT, offering additional information for clinical decision making regarding antithrombotic and anticoagulant therapy.

## Data Availability Statement

The raw data supporting the conclusions of this article will be made available by the authors, without undue reservation.

## Ethics Statement

The studies involving human participants were reviewed and approved by human ethics committee of Hangzhou First Hospital, Zhejiang University School of Medicine. The patients/participants provided their written informed consent to participate in this study.

## Author Contributions

KL: drafted/revised the manuscript, study concept or design, analysis or interpretation of data, acquisition of data, statistical analysis, and study supervision. LJ: drafted/revised the manuscript, study concept or design, analysis or interpretation of data, and acquisition of data. JR and WX: drafted/revised the manuscript, study concept or design, and analysis or interpretation of data. HH: acquisition of data and analysis or interpretation of data. GN: drafted/revised the manuscript. SY and CY: drafted/revised the manuscript, study concept or design, analysis or interpretation of data, contribution of vital reagents, tools, patients, study supervision, and obtained funding. All authors contributed to the article and approved the submitted version.

## Conflict of Interest

The authors declare that the research was conducted in the absence of any commercial or financial relationships that could be construed as a potential conflict of interest.
